# Computing and graphing probability values of pearson distributions: a SAS/IML macro

**DOI:** 10.1186/s13029-019-0076-2

**Published:** 2019-12-20

**Authors:** Qing Yang, Xinming An, Wei Pan

**Affiliations:** 1 Duke University, Durham, 27710 USA; 2 University of North Carolina at Chapel Hill, 27599, Chapel Hill, USA

**Keywords:** Pearson distributions, Curve fitting, Distribution-free statistics, Hypothesis testing

## Abstract

**Background:**

Any empirical data can be approximated to one of Pearson distributions using the first four moments of the data (Elderton WP, Johnson NL. Systems of Frequency Curves. 1969; Pearson K. Philos Trans R Soc Lond Ser A. 186:343–414 1895; Solomon H, Stephens MA. J Am Stat Assoc. 73(361):153–60 1978). Thus, Pearson distributions made statistical analysis possible for data with unknown distributions. There are both extant, old-fashioned in-print tables (Pearson ES, Hartley HO. Biometrika Tables for Statisticians, vol. II. 1972) and contemporary computer programs (Amos DE, Daniel SL. Tables of percentage points of standardized pearson distributions. 1971; Bouver H, Bargmann RE. Tables of the standardized percentage points of the pearson system of curves in terms of *β*_1_ and *β*_2_. 1974; Bowman KO, Shenton LR. Biometrika. 66(1):147–51 1979; Davis CS, Stephens MA. Appl Stat. 32(3):322–7 1983; Pan W. J Stat Softw. 31(Code Snippet 2):1–6 2009) available for obtaining percentage points of Pearson distributions corresponding to certain *pre-specified* percentages (or probability values; e.g., 1.0%, 2.5%, 5.0%, etc.), but they are little useful in statistical analysis because we have to rely on unwieldy second difference interpolation to calculate a probability value of a Pearson distribution corresponding to a given percentage point, such as an observed test statistic in hypothesis testing.

**Results:**

The present study develops a SAS/IML macro program to identify the appropriate type of Pearson distribution based on either input of dataset or the values of four moments and then compute and graph probability values of Pearson distributions for *any* given percentage points.

**Conclusions:**

The SAS macro program returns accurate approximations to Pearson distributions and can efficiently facilitate researchers to conduct statistical analysis on data with unknown distributions.

## Background

Most of statistical analysis relies on normal distributions, but this assumption is often difficult to meet in reality. Pearson distributions can be approximated for any data using the first four moments of the data [1–3]. Thus, Pearson distributions made statistical analysis possible for any data with unknown distributions. For instance, in hypothesis testing, a sampling distribution of an observed test statistic is usually unknown but the sampling distribution can be fitted into one of Pearson distributions. Then, we can compute and use a *p*-value (or probability value) of the approximated Pearson distribution to make a statistical decision for such distribution-free hypothesis testing.

There are both extant, old-fashioned in-print tables [4] and contemporary computer programs [5–9] that provided a means of obtaining percentage points of Pearson distributions corresponding to certain *pre-specified* percentages (or probability values; e.g., 1.0%, 2.5%, 5.0%, etc.). Unfortunately, they are little useful in statistical analysis because we have to employ unwieldy second difference interpolation for both skewness *√β*_1_ and kurtosis *β*_2_ to calculate a probability value of a Pearson distribution corresponding to a given percentage point, such as an observed test statistic in hypothesis testing. Thus, a new program is needed for efficiently computing probability values of Pearson distributions for *any* given data point; and therefore, researchers can utilize the program to conduct more applicable statistical analysis, such as distribution-free hypothesis testing, on data with unknown distributions.

Pearson distributions are a family of distributions which consist of seven different types of distributions plus normal distribution (Table 1). To determine the type of the Pearson distribution and the required parameters of the density function for the chosen type, the only thing we need to know is the first four moments of the data. Let *X* represent given data, and its first four central moments can be calculated by

1 $$ \left\{ \begin{array}{l} \mu_{1}'=E(X); \\ \mu_{i}=E[X-E(X)]^{i}=E[X-\mu_{1}']^{i}, i=2,3,4. \end{array} \right.  $$

**Table 1:** Types of Pearson distributions

Type	*κ*-Criterion	Density function	Domain
*Main Type*			
I	*κ*<0	$f(x)=y_{0}(1+\frac {x}{a_{1}})^{m_{1}}(1-\frac {x}{a_{2}})^{m_{2}}$	−*a*_1_≤*x*≤*a*_2_
IV	0<*κ*<1	$\phantom {\dot {i}\!}f(x)=y_{0}(1+\frac {x^{2}}{a^{2}})^{-m}e^{-\nu \arctan (x/a)}$	−*∞*<*x*<*∞*
VI	*κ*>1	$f(x)=y_{0}(x-a)^{q_{2}}x^{-q_{1}}\phantom {\dot {i}\!}$	*a*≤*x*<*∞*
*Transition Type*			
Normal	*κ*=0(*β*_2_=3)	$\phantom {\dot {i}\!}f(x)=y_{0}e^{-x^{2}/(2\mu _{2})}$	−*∞*<*x*<*∞*
II	*κ*=0(*β*_2_<3)	$f(x)=y_{0}(1-\frac {x^{2}}{a^{2}})^{m}$	−*a*≤*x*≤*a*
III	*κ*=±*∞*	$f(x)=y_{0}(1+\frac {x}{a})^{\gamma {a}}e^{-\gamma {x}}$	−*a*≤*x*<*∞*
V	*κ*=1	*f*(*x*)=*y*_0_*x*^−*p*^*e*^−*γ*/*x*^	0<*x*<*∞*
VII	*κ*=0(*β*_2_>3)	$f(x)=y_{0}(1+\frac {x^{2}}{a^{2}})^{-m}$	−*∞*<*x*<*∞*

The four central moments can also be uniquely determined by mean, variance, skewness, and kurtosis, which are more commonly used parameters for a distribution and easily obtained from statistical software. The relationships between skewness *√β*_1_ and the third central moment, and between kurtosis *β*_2_ and the fourth central moment are illustrated as follows:

2 $$ \left\{ \begin{array}{l} \surd\beta_{1}=\frac{\mu_{3}}{\mu_{2}^{3/2}} (also \beta_{1}=(\surd\beta_{1})^{2}=\frac{\mu_{3}^{2}}{\mu_{2}^{3}}); \\ \beta_{2}=\frac{\mu_{4}}{\mu_{2}^{2}}. \end{array} \right.  $$

Once the four central moments or the mean, variance, skewness, and kurtosis are calculated, the types of Pearson distributions to which *X* will be approximated can be determined by a *κ*-criterion that is defined as follows [1]:

3 $$ \kappa=\frac{\beta_{1}(\beta_{2}+3)^{2}}{4(4\beta_{2}-3\beta_{1})(2\beta_{2}-3\beta_{1}-6)}.   $$

The determination of types of Pearson distributions by the *κ*-criterion (Eq. 3) is illustrated in Table 1. From Table 1, we can also see that for each type of Pearson distributions, its density function has a closed form with a clearly defined domain of *X*. The closed form of density functions made numerical integration possible for obtaining probability values of approximated Pearson distributions. For each type of Pearson distributions, the required parameters of the density function are calculated by using different formulas. Without loss of generality, we illustrate the type IV formula below. The formula for the rest of the types can be retrieved from [1].

The density function for type IV Pearson distribution is

4 $$  y = y_{0}\left(1+\frac{(x-\lambda)^{2}}{a^{2}}\right)^{-m}e^{-\nu\tan^{-1}(x-\lambda)/a},  $$

where $m=\frac {1}{2}(r+2)$, $\nu =\frac {-r(r-2)\sqrt \beta _{1}}{\sqrt {16(r-1)-\beta _{1}(r-2)^{2}}}$, $r=\frac {6(\beta _{2}-\beta _{1}-1)}{2\beta _{2}-3\beta _{1}-6}$, the scale parameter $a=\sqrt {(\mu _{2}/16)}\sqrt {(16(r-1)-\beta _{1}(r-2)^{2})}$, the location parameter *λ*=*μ*_1_+*νa*/*r*, and normalization coefficient $y_{0}=\frac {N}{aF(r,\nu)}$.

The required parameters for each type of Pearson distribution density functions will be automatically computed in a SAS/IML [10] macro program described in the next section. Then, probability values of Pearson distributions can be obtained through numerical integration with the SAS subroutine QUAD.

## Implementation

To add the flexibility to the macro, we allow two different ways to input required information. The first one is to input the dataset and variable. The macro will automatically calculate the mean, variance, skewness, and kurtosis of the input variable. The second one is to input the mean, variance, skewness, and kurtosis of the variable directly. The main SAS/IML macro program (see Additional file 1) to compute and graph probability values of Pearson distributions is as follows: %PearsonProb(data=, var=, mean=, variance=, skew=, kurt=, x0=, plot=)

wheredata = the name of the dataset to calculate four moments (this input can be omitted if mean, variance, skewness, and kurtosis input used); var = the name of variable in the dataset to calculate moments (this input can be omitted if mean, variance, skewness, and kurtosis input used); mean = the mean of the variable (this input can be omitted if data and var input used); variance = the variance of the variable (this input can be omitted if data and var input used); skew = the skewness of the variable (this input can be omitted if data and var input used); kurt = the kurtosis of the variable (this input can be omitted if data and var input used); x0 = the percentage point *x*_0_; plot = 1 for graph, 0 for no graph.

**Fig. 1:**
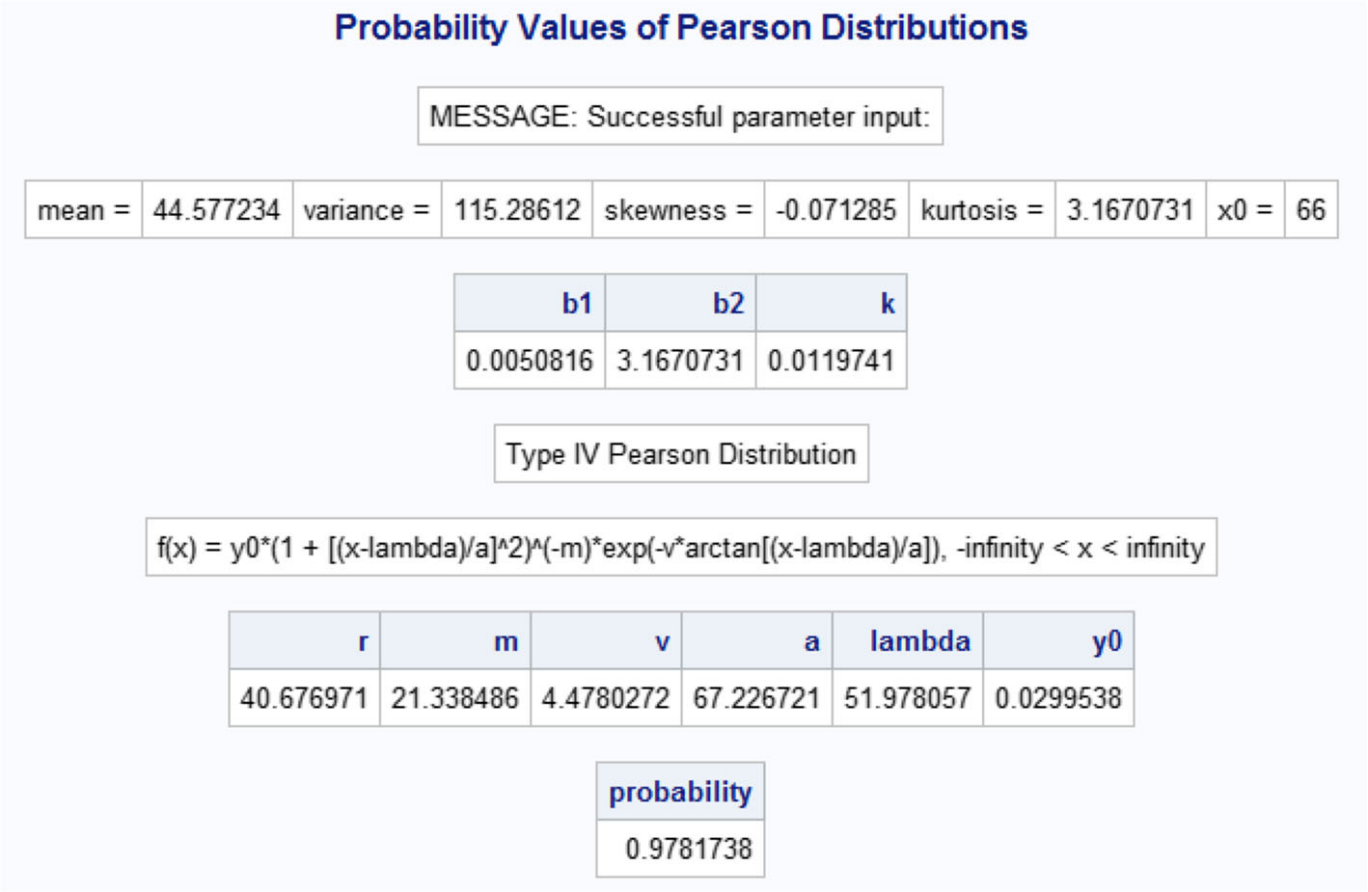
SAS output for Type IV Pearson distribution parameters and probability

This SAS/IML macro program has four steps. The first step is to either calculate mean, variance, skewness, and kurtosis based on the input dataset or take the four values directly from inputted parameters. The second step is to calculate *κ* by using Eq. (3) and identify a specific type of Pearson distribution based on the *κ*-criterion displayed in Table 1. Once the type of Pearson distribution is determined, in the third step, the macro will calculate the parameters of density function for the specific type of Pearson distribution. For example, for type IV Pearson distribution, *y*_0_, *m*, *ν*, *a*, and *λ* will be calculated according to the specifications underneath Eq. (4). In the fourth and last step, the probability value of the specific type of Pearson distribution corresponding to the inputted percentage point *x*_0_ will be calculated by the SAS subroutine QUAD for numerical integration. If the inputted *x*_0_ is beyond the defined domain, a warning message will be printed as “WARNING: x0 is out of the domain of type VI Pearson distribution,” for example. If successful, the computed probability value along with the parameters are printed (see Fig. 1).

To graph the probability value on the approximated density function of the Pearson distribution, a small SAS/IML macro %plotprob was written for use within the main SAS/IML macro %PearsonProb(data=, var=, mean=, variance=, skew=, kurt=, x0=, plot=). If 1 is inputted for plot, the SAS subroutines GDRAW, GPLOY, etc. are called in the small graphing macro for plotting the density function and indicating probability value. Otherwise (i.e., plot = 0), no graph is produced.

To illustrate the process, we provide an example of input and output below (two example datasets are available online: Additional files 2 & 3). One could either input a dataset and variable name (Item 1) or input the values of “mean”, “variance”, “skewness”, and “kurtosis” (Item 2) to the %PearsonProb macro. Both the dataset “dataIV” and the values of the four moments for this example are taken from [1].

1. %PearsonProb(data = pearson.dataIV, var = x, x0 = 66, plot = 1);
2. %PearsonProb(mean = 44.578, variance = 115, skew = 0.07325, kurt = 3.1729, x0 = 66, plot = 1).

**Fig. 2:**
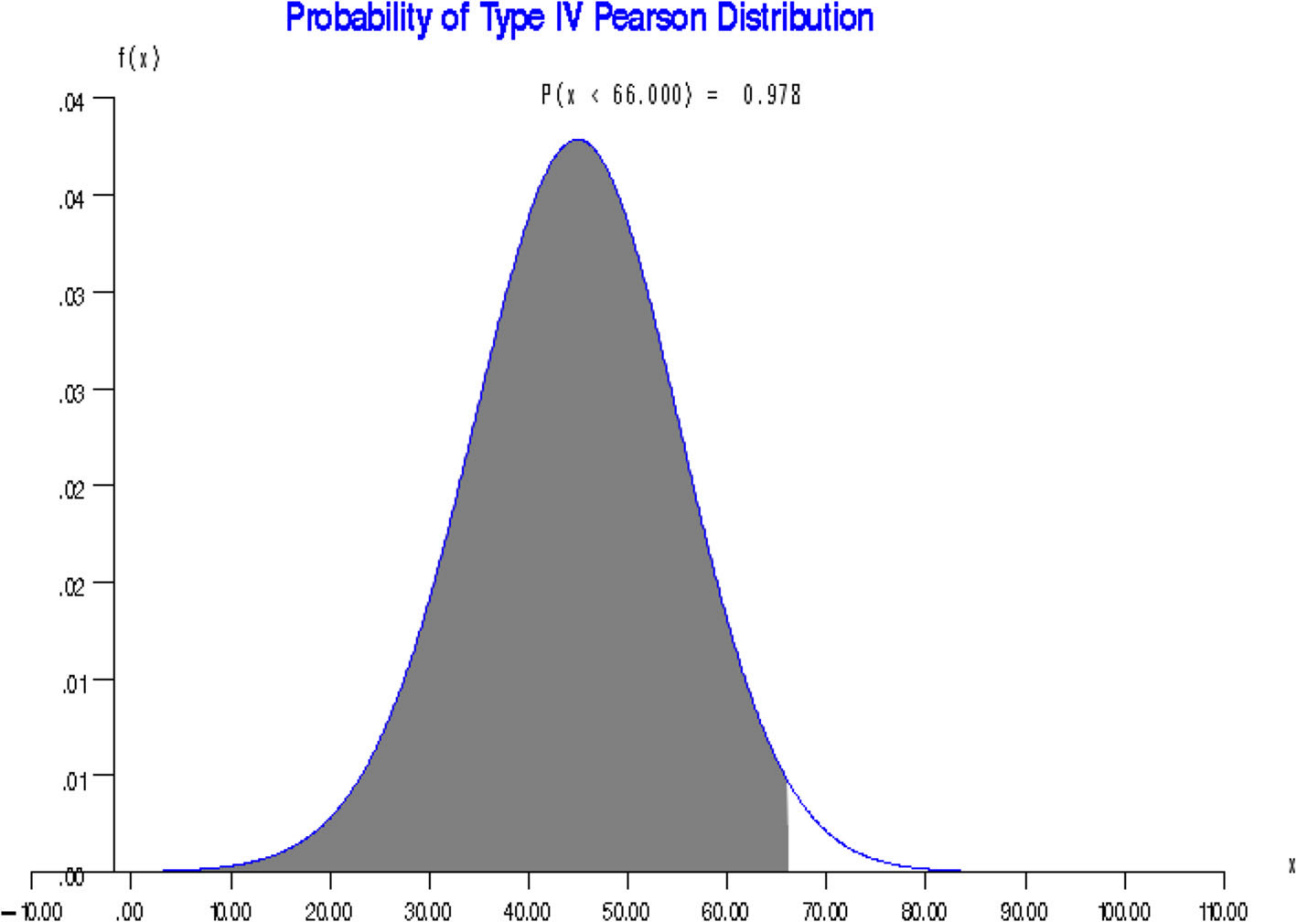
A type IV Pearson distribution with a probability value indicated

The outputs from both the statements are the same. The standard output (see Fig. 1) includes the values of mean, variance, skewness, and kurtosis; and indicates the type of the Pearson distribution identified. It also outputs the formula for the density function and the values of the parameters of the density function. Lastly, it prints the calculated probability. Since we used the plot = 1 option, a figure to illustrate the distribution and probability is also produced (see Fig. 2).

## Results

To evaluate the accuracy of the SAS/IML macro program for computing and graphing probability values of Pearson distributions, the calculated parameters of the approximated Pearson distributions from this SAS/IML macro were first compared with the corresponding ones in [1]. As can be seen in Table 2, the absolute differences between the calculated parameters from the SAS/IML macro and those from [1]’s tables are all very small with almost all of them less than.001 and a few less than.019. The same story applies to the relative differences with an unsurprising exception (4.46%) of *κ* for type IV whose original magnitude is very small.

**Table 2:** Computed parameters and their accuracy

		Value from	Value from Elderton	Absolute Difference^b^	Relative Difference^c^
Type^a^	Parameter	SAS/IML Macro	and Johnson (1969)		
I	*β*_1_	.507296	.507296	<.0001	<.01%
	*β*_2_	2.935111	2.935110	<.0001	<.01%
	*κ*	-.264690	-.264500	.0002	.07%
	*r*	5.186821	5.186811	<.0001	<.01%
	*α*_1_	1.977543	1.996380	.0188	.94%
	*α*_2_	13.508428	13.527280	.0189	.14%
	*m*_1_	.406954	.409833	.0029	.70%
	*m*_1_	2.779867	2.776878	.0030	.12%
IV	*β*_1_	.005366	.005366	<.0001	<.01%
	*β*_2_	3.172912	3.172912	<.0001	<.01%
	*κ*	.012230	.012800	.0006	4.46%
	*r*	39.442562	39.442540	<.0001	<.01%
	*v*	4.388796	4.388794	<.0001	<.01%
	*α*	13.111988	13.111980	<.0001	<.01%
	*m*	20.721280	20.721270	<.0001	<.01%
VI	*β*_1_	.995360	.995361	<.0001	<.01%
	*β*_2_	4.739349	4.739349	<.0001	<.01%
	*κ*	1.894437	1.895000	.0006	.03%
	*r*	-33.421430	-33.421290	.0001	<.01%
	*q*_1_	42.030520	42.030800	.0003	<.01%
	*q*_2_	6.609095	6.609500	.0004	<.01%
	*α*	10.379832	10.379470	.0004	<.01%

^a^Elderton and Johnson (1969) does not have the other types of Pearson distributions

^b^Absolute Difference = |Value from Elderton and Johnson (1969) − Value from SAS/IML Macro |

^c^Relative Difference = |(Value from Elderton and Johnson (1969) − Value from SAS/IML Macro)/Value from Elderton and Johnson (1969) |×100%

Then, the computed probability values from the SAS/IML macro were evaluated using the percentage points in [4]’s Table 32 (p. 276) corresponding to probability values of 2.5% and 97.5% for illustration purposes only. From Table 3, we can see that the probability values computed from the SAS/IML macro are very close to.025 (or 2.5%) and.975 (or 97.5%), respectively, with a high degree of precision (less than.0001).

**Table 3:** Computed probability values and their accuracy

			Percentage Point from Pearson and Hartley (1972)		Probability Value from SAS/IML Macro		Absolute Difference^b^	
Type^a^	*√β*_1_	*β*_2_	For 2.5%	For 97.5%	2.5%	97.5%	For 2.5%	For 97.5%
Normal	.0	3.0	-1.9600	1.9600	.0249970	.9750020	<.00001	<.00001
I	.6	3.2	-1.5998	2.2320	.0249965	.9749989	<.00001	<.00001
II	.0	2.6	-1.9196	1.9196	.0250030	.9749970	<.00001	<.00001
IV	1.4	8.6	-1.5068	2.3801	.0249838	.9749471	.00002	.00005
VI	2.0	11.2	-1.1915	2.5545	.0250054	.9750021	.00001	<.00001
VII	.0	8.4	-1.9925	1.9925	.0249999	.9750001	<.00001	<.00001

^a^Pearson and Hartley (1972) does not have examples of types III and V

^b^Absolute Difference = |.025 − Probability value from SAS/IML macro |; and = |.975 − Probability value from SAS/IML macro |, respectively

## Discussion

Pearson distributions are a family of non-parametric distributions. It is often used when the normal distribution assumption is not applicable to the data. In this paper, the first approach of inputting dataset as parameters for the macro is more often used. The second approach of entering first four moments as parameters are more helpful when the researcher already performed some descriptive statistics based on the data in the first approach.

## Conclusions

The new SAS/IML macro program provides an efficient and accurate means to determine the type of Pearson distribution based on either a dataset or values of the first four moments and then compute probability values of the specific Pearson distributions. Thus, researchers can utilize this SAS/IML macro program in conducting distribution-free statistical analysis for any data with unknown distributions. The SAS/IML macro program also provides a nice feature of graphing the probability values of Pearson distributions to visualize the probability values on the Pearson distribution curves.

## Availability and requirements

**Project name**: PearsonProb

**Project home page**: To be available

**Operating system(s)**: Platform independent

**Programming language**: SAS/IML

**Other requirements**: SAS 9.4 or higher

**License**: Not applicable

**Any restrictions to use by non-academics**: None

## Additional material

Additional file 1 SAS/IML macro program. The SAS/IML macro program for computing and graphing probability values of Pearson distributions is available as an additional file, *PearsonDistributionProbfinal.sas*

Additional file 2 Sample dataset 1. The dataset *dataI.sas7bdat* was taken from [1].

Additional file 3 Sample dataset 2. The dataset *dataIV.sas7bdat* was taken from [1].

## Data Availability

Not applicable.

## References

[1] Elderton WP, Johnson NL (1969). Systems of Frequency Curves.

[2] Pearson K (1895). Contributions to the mathematical theory of evolution. ii. skew variations in homogeneous material. Philos Trans R Soc Lond Ser A.

[3] Solomon H, Stephens MA (1978). Approximations to density functions using pearson curves. J Am Stat Assoc.

[4] Pearson ES, Hartley HO (1972). Biometrika Tables for Statisticians, vol. II.

[5] Amos DE, Daniel SL (1971). Tables of percentage points of standardized pearson distributions, Research Report SC-RR-71 0348.

[6] Bouver H, Bargmann RE (1974). Tables of the standardized percentage points of the pearson system of curves in terms of *β*_1_ and *β*_2_, Technical Report No. 107.

[7] Bowman KO, Shenton LR (1979). Approximate percentage points for pearson distributions. Biometrika.

[8] Davis CS, Stephens MA (1983). Approximate percentage points using pearson curves. Appl Stat.

[9] Pan W (2009). A SAS/IML macro for computing percentage points of pearson distributions. J Stat Softw.

[10] SAS Institute Inc.SAS/IML 9.3 User’s Guide. 2011. http://www.sas.com/. Accessed 23 Jun 2012.

